# Efficacy of a four-tier infection response system in the emergency department during the coronavirus disease-2019 outbreak

**DOI:** 10.1371/journal.pone.0256116

**Published:** 2021-08-12

**Authors:** Arom Choi, Ha Yan Kim, Ara Cho, Jiyoung Noh, Incheol Park, Hyun Soo Chung

**Affiliations:** 1 Department of Emergency Medicine, Yonsei University College of Medicine, Seoul, South Korea; 2 Department of Biomedical Systems Informatics, Biostatistics Collaboration Unit, Yonsei University College of Medicine, Seoul, South Korea; 3 Center for Disaster Relief, Training, and Research, Severance Hospital, Yonsei University College of Medicine, Seoul, South Korea; San Giuseppe Hospital, ITALY

## Abstract

**Introduction:**

The coronavirus disease (COVID-19) pandemic has delayed the management of other serious medical conditions. This study presents an efficient method to prevent the degradation of the quality of diagnosis and treatment of other critical diseases during the pandemic.

**Methods:**

We performed a retrospective observational study. The primary outcome was ED length of stay (ED LOS). The secondary outcomes were the door-to-balloon time in patients with suspected ST-segment elevation myocardial infarction and door-to-brain computed tomography time for patients with suspected stroke. The outcome measures were compared between patients who were treated in the red and orange zones designated as the changeable isolation unit and those who were treated in the non-isolation care unit. To control confounding factors, we performed propensity score matching, following which, outcomes were analyzed for non-inferiority.

**Results:**

The mean ED LOS for hospitalized patients in the isolation and non-isolation care units were 406.5 min (standard deviation [SD], 237.9) and 360.2 min (SD, 226.4), respectively. The mean difference between the groups indicated non-inferiority of the isolation care unit (p = 0.037) but not in the patients discharged from the ED (p>0.999). The mean difference in the ED LOS for patients admitted to the ICU between the isolation and non-isolation care units was -22.0 min (p = 0.009). The mean difference in the door-to-brain computed tomography time between patients with suspected stroke in the isolation and non-isolation care units was 7.4 min for those with confirmed stroke (p = 0.013), and -20.1 min for those who were discharged (p = 0.012). The mean difference in the door-to-balloon time between patients who underwent coronary angiography in the isolation and non-isolation care units was -2.1 min (p<0.001).

**Conclusions:**

Appropriate and efficient handling of a properly planned ED plays a key role in improving the quality of medical care for other critical diseases during the COVID-19 outbreak.

## Introduction

The coronavirus disease (COVID-19) outbreak, which started at the end of 2019 and is caused by severe acute respiratory syndrome coronavirus 2, has become a major issue that could disrupt the entire health care system in most countries. Currently, most medical resources are focused on the diagnosis and treatment of COVID-19, which hinders the management of patients requiring emergency care for other chronic and critical diseases [1, 2].

The emergency department (ED) plays an important role in the diagnosis and management of life-threatening diseases. However, when fever or respiratory symptoms accompany other symptoms or serious diseases irrelevant to COVID-19, the current setup of medical resource distribution may delay the management of these medical conditions, resulting in serious disability or mortality [3–6].

According to the *Morbidity and Mortality Weekly Report* by the Centers for Disease Control and Prevention, visits to EDs in the United States during the current COVID-19 pandemic have decreased by 42%, whereas heart attacks and strokes diagnosed in EDs during the first half of 2020 decreased by 23% and 20%, respectively [7]. Diegoli et al. reported a marked decrease in hospital admissions for stroke and expressed concerns regarding the poor quality of the treatment of health conditions other than COVID-19 due to social restrictions and lack of resources [8].

Various recent studies have demonstrated how to efficiently respond to the COVID-19 pandemic by changing the structure of the ED [9–12]. However, these studies have focused on the ED design and resource placement for the treatment of confirmed COVID-19 cases without missing any case. It is crucial that the quality of diagnosis and treatment in the ED, the first gateway to emergency care, be maintained. Therefore, this study presents a novel and efficient method of care that involves hardware and software modification to prevent the degradation of the quality of the diagnosis and treatment of critical diseases, notwithstanding the challenges of the COVID-19 pandemic.

## Materials and methods

This study was conducted according to the Strengthening the Reporting of Observational Studies in Epidemiology guidelines [13], and was approved by the institutional review board of our hospital (approval number 4-2020-0919). The requirement for informed consent was waived, and the study protocol complied with the tenets of the Declaration of Helsinki.

### Study design and setting

This retrospective study was conducted in the level 1 ED of a tertiary teaching hospital using data collected prospectively. A female patient who arrived in South Korea from China was the first confirmed case of COVID-19 in South Korea. Her case was confirmed on January 20, 2020, which was 21 days after China reported “an unidentified viral pneumonia from Wuhan, Hubei” to the World Health Organization on December 31, 2019. In 2019, before the COVID-19 pandemic, an average of 8,738 patients visited our ED in a month. Approximately 21.1% of those patients were admitted to the hospital from the ED. The number of patients admitted to intensive care units (ICU), including the medical ICU, surgical ICU, stroke unit, cardiac care unit, and neuro-critical care unit, was an average of 200 per month. In our hospital, the setup of the ED was redesigned sequentially to effectively control the infection from February 2020 onward, which was after the first COVID-19 case was recorded in South Korea. Thus, this study was conducted between March 1, 2020, and May 31, 2020, and all patients aged >17 years who visited the ED during this period were enrolled. Patients who visited for reasons other than medical care, those who had missing data, and those who were discharged immediately after they visited the fever clinic were excluded.

### Data collection

Data were automatically collected using a clinical research analysis portal system at the medical information department of the hospital. With this system, we could use random identification numbers to anonymously extract clinical data, including the variables and conditions selected for research. Data on the following variables of all patients who visited the ED during the study period were extracted: age; sex; vital signs such as systolic blood pressure (SBP), diastolic blood pressure (DBP), heart rate (HR), body temperature, and respiratory rate; medical history such as a history of hypertension, diabetes mellitus, tuberculosis, hepatitis, cancer, pulmonary disease, cardiovascular disease, renal disease, cerebrovascular disease, and organ transplantation; Korean Triage and Acuity Scale (KTAS) score; time of ED visit; time of disposition and discharge; disposition information such as admission to the general ward, ICU, or isolation wards, and discharge; interventions such as central line insertion, use of inotropes, mechanical ventilation, and continuous renal replacement therapy; door-to-brain computed tomography (CT) time for patients suspected to have a stroke; and door-to-balloon time for patients suspected to have ST-segment elevation myocardial infarction.

### Post-COVID-19 institutional setting of the emergency department

#### Screening for suspected COVID-19 cases in the advanced ED triage unit

Before the COVID-19 outbreak, the triage unit of our hospital was located inside the ED. Patients were checked for vital signs and severity of illness by a qualified triage nurse. After the COVID-19 outbreak, we moved the triage unit forward to prevent in-hospital spread of infection among patients and medical personnel by preventing patients with fever or respiratory symptoms from entering the ED. All patients and their companions were screened in the advanced ED triage unit, regardless of the purpose of their visit. The advanced ED triage was equipped with high-efficiency particulate absorbing-filters, and all triage nurses wore full Level-D personal protective equipment (PPE), which comprised N95 masks, eye shields, disposable gloves, caps, and gowns. Trained triage nurses screened patients using broadened case definitions for suspected COVID-19 cases. The screening criteria were as follows: fever of >37.5°C, respiratory symptoms such as cough, sputum, rhinorrhea, sore throat, dyspnea, or loss of senses of smell and taste, travel history, history of contact with a patient with confirmed COVID-19, history of visit to an area with community spread of COVID-19, known history or confirmed diagnosis of pneumonia, recently developed or aggravated symptoms in 2 weeks, and vital signs. We set up a waiting area for each patient outside the ED in case of a sudden influx of patients into the ED.

#### Four-tier system for spatial strategy

**Fig 1:**
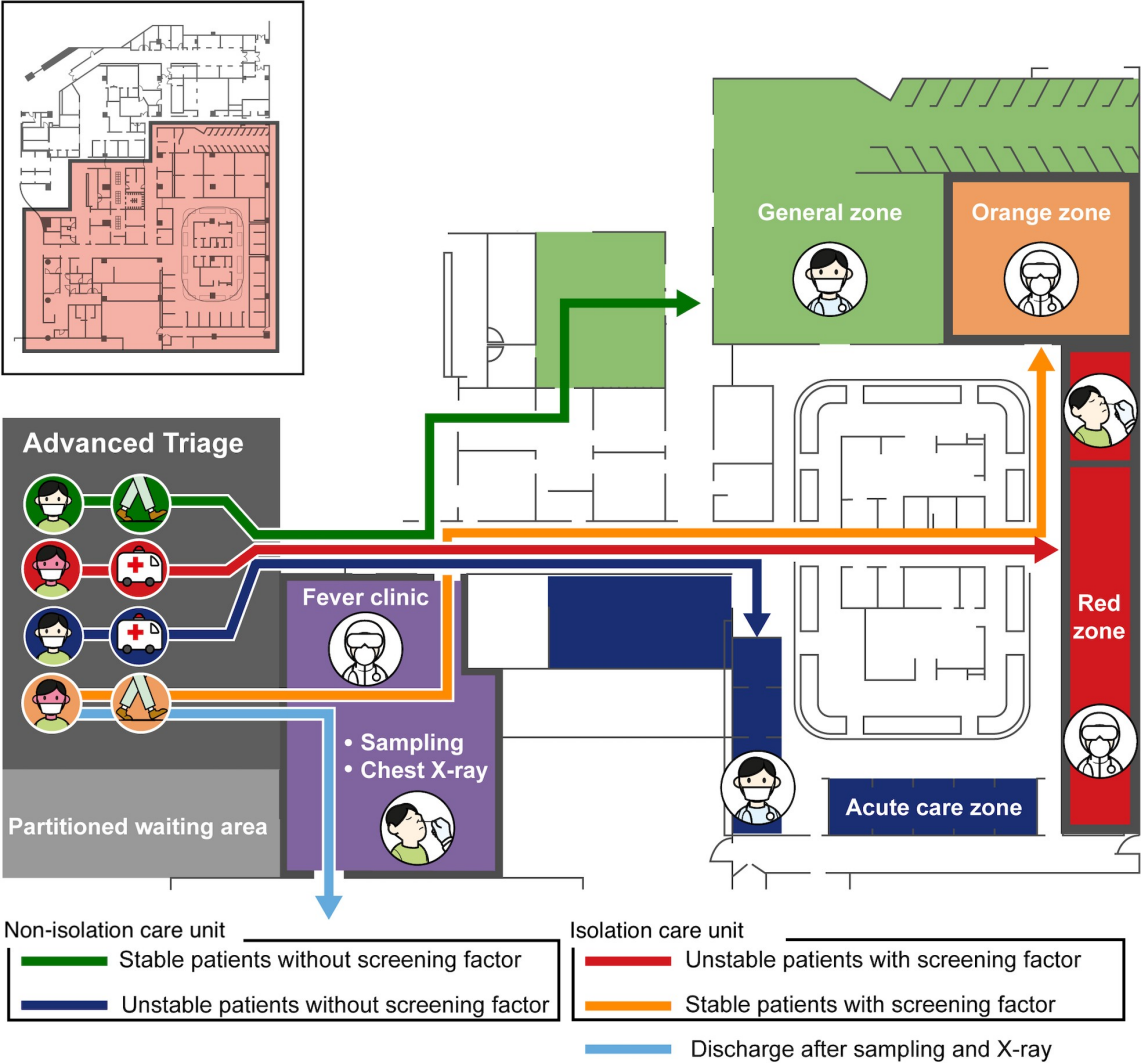
Geometric map of the emergency department showing the four-tier infection response system.

We compartmentalized the emergency room into four sections, comprising the isolation care unit as the red and orange zones, emergency fever clinic, acute-care unit, and general zone, to separate the spaces intended for treating patients with suspected infection from those intended for treating patients without any sign of infection (Fig 1). A unidirectional air-conditioning system was installed in this space and a dividing wall was installed between all the beds in the ED. A radius of 2 m was maintained while transferring patients. Furthermore, an acrylic partition was installed around the working area of the medical personnel to block infection caused by droplets.

All the patients who entered the ED were separated and monitored. Patients who met the screening criteria, had any sign of infection or confirmed pulmonary consolidation on chest X-rays, or needed urgent resuscitation due to unstable vital signs were transferred to the red zone for further evaluation and initiation of treatment. Stable patients who met the screening criteria were transferred to the orange zone. Patients who did not meet these screening criteria were cared for as before in the general zone.

The emergency fever clinic, which was set up in the isolation walking unit, was established in a negative pressure unit at the entrance of the ED. All medically stable patients who met the screening criteria and were not bedridden met a doctor in this fever clinic. Based on the medical conditions and chest X-ray results of the patients, the doctor examined and assigned those who needed further evaluation and treatment to the red or orange zone. If the patient did not need any additional evaluation, the doctor provided the prescription for oral medication or ordered discharge immediately after taking respiratory samples for a COVID-19 test and a chest X-ray. All patients who met the screening criteria in the advanced ED triage unit were tested for COVID-19; their respiratory samples were tested for severe acute respiratory syndrome coronavirus 2 in the fever clinic or in the ED using the reverse transcription-polymerase chain reaction test. The diagnostic department of our hospital ran COVID-19 tests a total of six times a day, at 4-h intervals. Patients who were confirmed as COVID-19 negative were moved to the general zone to be treated continuously, and the red and orange zone were sterilized and prepared for patients with suspected infection every four hours. All the medical personnel who worked in the fever clinic followed the hospital regulations for infection control, such as the use of PPE. If a confirmed case of COVID-19 was recorded within the ED, the patient was treated in a large negative pressure room of the fever clinic.

#### Operation of the COVID-19 ward and the preemptive isolation ward

Two wards that consisted entirely of negative pressure rooms and their antechambers were used as the COVID-19 ward and preemptive isolation ward. One ward with six beds was used as a ward for patients with confirmed COVID-19 who needed critical care, and the other one with nine beds was used as a preemptive isolation ward where patients whose COVID-19 test results were not yet confirmed could be treated.

#### Software modification of the emergency department

All aerosol-generating procedures, such as oral and endotracheal suction and application of a nebulizing inhaler, were performed in the isolation room, and PPEs with powered air-purifying respirators were used during high-risk procedures such as endotracheal intubation. The “code ICE” was established for the ED to evacuate a path when patients in the red or orange zone were transferred for a CT scan, X-ray, or to the ward for admission. If code ICE was announced in the ED, PPE-equipped safety personnel evacuated the path and prevented movement of people in the ED.

Wearing of KF-94 masks, which is an N95 respirator equivalent, and disposable gowns was required in the general zone to prevent contact with asymptomatic patients with COVID-19. Moreover, the medical team of the ED was regularly trained on infection control and PPE use. Activity guidance and tracking of movement and physical contact were performed retroactively by the epidemiological team to determine whether other patients or staff in the ED were exposed, and active and manual monitoring were conducted for potentially exposed patients and staff based on the degree of contact with confirmed patients.

### Intervention and outcome variables

The intervention was an admission of screened patients into the isolation care unit (the red and orange zone). Primary outcomes were duration from the time of admission into the ED to the time of hospitalization or discharge from the ED and time of admission into the ICU. Secondary outcomes were the door-to-balloon time for patients who were suspected to have ST-segment elevation myocardial infarction and underwent coronary angiography and door-to-brain CT time for patients suspected to have an ischemic or hemorrhagic stroke.

### Statistical analyses

Categorical variables were reported as counts and percentages, and continuous variables were expressed as mean and standard deviation. SBP, DBP, and HR were each categorized into three groups: SBP into <100 mmHg, >180 mmHg, or others; DBP into <60 mmHg, >140 mmHg, or others; and HR into <60 bpm, >100 bpm, or others. To control confounding factors, we performed propensity score matching using SAS One-To-Many Macro; starting with the eighth decimal digit to the first digit, the nearest neighbor was matched 1:1. The variables used in the matching were selected with reference to previous studies. Before matching, continuous variables were analyzed using the independent t-test, whereas categorical variables were analyzed using the chi-square test. After matching, continuous variables were analyzed using the paired t-test, and categorical variables were analyzed using the McNemar’s test or generalized estimating equation. Since the data were paired through propensity score matching, it is appropriate to compare in pairs when comparing groups. The Kolmogorov−Smirnov test was used to test for normality in the two groups. Outcomes were analyzed for non-inferiority; the clinically determined non-inferiority margin was 60 min. Additionally, we evaluated the 95% confidence interval (CI) with bootstrapping (10,000 times resampling with replacement) for internal validation using R package, version 3.4.3 (http://www.R-project.org). Differences were considered statistically significant at p<0.05. All statistical analyses were performed using SAS software version 9.4 (SAS Institute, Cary, NC, USA).

## Results

### Patient demographics and propensity score matching

**Fig 2:**
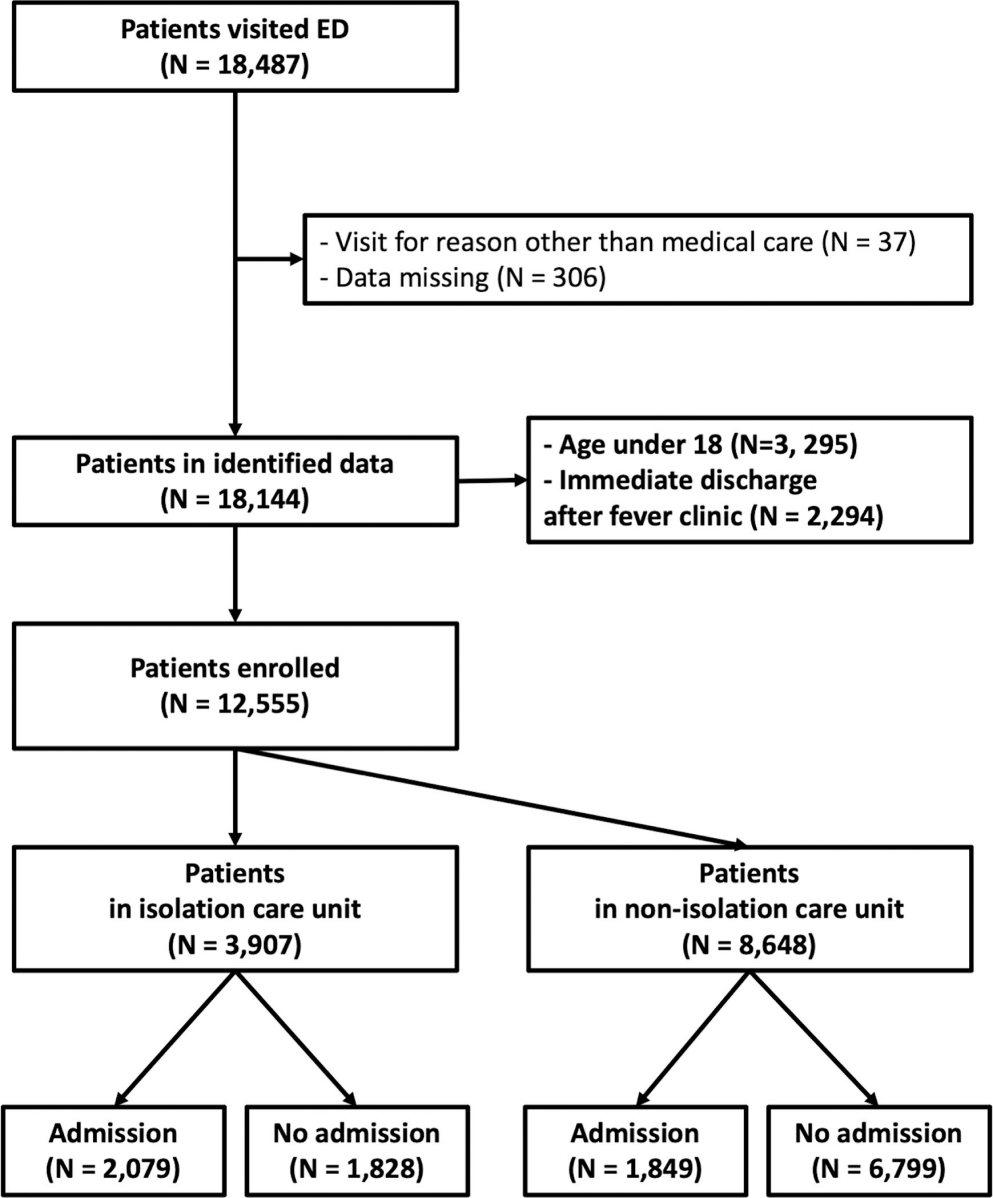
Flow chart of the study, ED: Emergency department.

In total, 18,487 patients visited the ED between March 1, 2020, and May 31, 2020. We excluded 306 patients with missing data and 37 patients who visited the ED for non-medical purposes. Furthermore, 3,295 patients aged <18 years and 2,294 patients who were discharged immediately after their samples were taken for the reverse transcription-polymerase chain reaction test, after their chest X-ray exams, and after simple treatment for chief complaints were excluded as well. Among the 12,555 enrolled patients, 8,648 (68.9%) patients were admitted into the non-isolation care unit (the acute-care unit and the general zone), and 3,907 (31.1%) patients who met the screening criteria were admitted into the isolation care unit (the red or orange zone). The rate of admission to the hospital via the ED was an average of 31.3% for 3 months, which was higher than the admission rate (21.1%) for 2019 (Fig 2).

Propensity score matching was performed for 1,346 hospitalized patients and 1,683 discharged patients. The baseline demographics and variables that were used for matching in each group are shown in Tables 1 and 2. Before matching, the absolute standardized difference (ASD) between the patient group admitted into the isolation care unit (red or orange zone) and to the non-isolation care unit was >20% for the following variables, resulting in a statistically significant difference: age, DBP, HR, KTAS, central catheterization, and use of inotropes. For patients discharged from the ED, the ASD was >20% for the following variables: age, DBP, KTAS, history of cancer, and pulmonary disease. After propensity score matching, the ASDs of all variables were within 20%, implying that each variable was balanced appropriately between the two groups.

**Table 1:** Propensity score matching for hospitalized patients.

	Before matching				After matching			
	General	Red/Orange	p-value	ASD	General	Red/Orange	p-value	ASD
	N = 1849	N = 2079			N = 1346	N = 1346		
**Age**	60.4±17.8	65.0±17.6	<0.001	26.43	62.3±17.6	62.9±18.7	0.287	3.44
**Sex (male)**	1005(54.4)	1142(54.9)	0.717	1.16	736(54.7)	732(54.4)	0.862	0.60
**Systolic blood pressure**			<0.001	18.75			0.797	5.27
1(<100)	168(9.1)	319(15.3)			143(10.6)	137(10.2)		
2(100–180)	1564(84.6)	1654(79.6)			1124(83.5)	1131(84.0)		
3(> = 180)	117(6.3)	106(5.1)			79(5.9)	78(5.8)		
**Diastolic blood pressure**			<0.001	30.07			0.733	14.51
1(<60)	152(8.2)	379(18.2)			140(10.4)	145(10.8)		
2(60–140)	1693(91.6)	1695(81.5)			1203(89.4)	1198(89.0)		
3(> = 140)	4(0.2)	5(0.2)			3(0.2)	3(0.2)		
**Heart rate**			<0.001	50.41			0.590	0
1(<60)	108(5.8)	65(3.1)			58(4.3)	60(4.5)		
2(60–100)	1313(71.0)	1061(51.0)			874(64.9)	881(65.5)		
3(> = 100)	428(23.2)	953(45.8)			414(30.8)	405(30.1)		
**KTAS**			<0.001	24.16			0.099	7.45
1	43(2.3)	101(4.9)			38(2.8)	42(3.1)		
2	303(16.4)	416(20.0)			215(16.0)	232(17.2)		
3	751(40.6)	840(40.4)			527(39.2)	543(40.3)		
4	655(35.4)	660(31.8)			505(37.5)	473(35.1)		
5	97(5.3)	62(3.0)			61(4.5)	56(4.2)		
**Hypertension**	764(41.3)	916(44.1)	0.081	5.58	579(43.0)	572(42.5)	0.768	1.05
**Diabetes mellitus**	439(23.7)	630(30.3)	<0.001	14.81	353(26.2)	340(25.3)	0.548	2.21
**Tuberculosis**	50(2.7)	96(4.6)	0.002	10.20	42(3.1)	41(3.1)	0.909	0.43
**Hepatitis carrier**	106(5.7)	78(3.8)	0.003	9.33	66(4.9)	60(4.5)	0.574	2.11
**Cancer**	668(36.1)	949(45.7)	<0.001	19.45	562(41.8)	550(40.9)	0.595	1.81
**Pulmonary disease**	94(5.1)	203(9.8)	<0.001	17.93	85(6.3)	81(6.0)	0.735	1.24
**Cardiovascular disease**	22(15.8)	374(18.0)	0.067	5.87	221(16.4)	222(16.5)	0.957	0.20
**Renal disease**	164(8.9)	236(11.4)	0.010	8.24	142(10.6)	137(10.2)	0.750	1.22
**Cerebrovascular disease**	129(7.0)	164(7.9)	0.277	3.48	102(7.6)	100(7.4)	0.883	0.56
**Organ transplantation**	57(3.1)	81(3.9)	0.167	4.43	48(3.6)	47(3.5)	0.915	0.40
**Central catheterization**	54(2.9)	197(9.5)	<0.001	27.44	54(4.0)	57(4.2)	0.745	1.12
**Mechanical ventilation**	49(2.7)	96(4.6)	0.002	10.53	42(3.1)	46(3.4)	0.669	1.67
**Continuous renal replace**	2(0.1)	5(0.2)	0.458	3.17	1(0.1)	1(0.1)	>0.999	0
**Inotropic use**	116(6.3)	360(17.3)	<0.001	34.75	114(8.5)	109(8.1)	0.695	1.35

Variables are shown as mean ± standard deviation or number (percentage).

Abbreviations: ASD, absolute standardized difference; KTAS, Korean Triage and Acuity Scale

**Table 2:** Propensity score matching for discharged patients.

	Before matching				After matching			
	General	Red/Orange	p-value	ASD	General	Red/Orange	p-value	ASD
	N = 6799	N = 1828			N = 1683	N = 1683		
**Age**	49.9±19.3	56.0±20.6	<0.001	30.31	53.6±19.8	54.8±20.6	0.011	6.11
**Sex (male)**	3178(46.7)	791(43.3)	0.008	6.98	737(43.8)	727(43.2)	0.539	1.20
**Systolic blood pressure**			<0.001	11.34			0.097	9.50
1(<100)	196(2.9)	94(5.1)			78(4.6)	71(4.2)		
2(100–180)	6249(91.9)	1619(88.6)			1519(90.3)	1504(89.4)		
3(> = 180)	354(5.2)	115(6.3)			86(5.1)	108(6.4)		
**Diastolic blood pressure**			<0.001	25.47			0.451	14.72
1(<60)	203(3.0)	156(8.5)			115(6.8)	107(6.4)		
2(60–140)	6590(96.9)	1669(91.3)			1567(93.1)	1573(93.5)		
3(> = 140)	6(0.1)	3(0.2)			1(0.1)	3(0.2)		
**Heart rate**			<0.001	47.41			0.501	8.35
1(<60)	257(3.8)	38(2.1)			24(1.4)	32(1.9)		
2(60–100)	5455(80.2)	1132(61.9)			1101(65.4)	1100(65.4)		
3(> = 100)	1087(16.0)	658(36.0)			558(33.2)	551(32.7)		
**KTAS**			<0.001	38.35			0.498	4.16
1	15(0.2)	41(2.2)			13(0.8)	14(0.8)		
2	458(6.7)	181(9.9)			147(8.7)	159(9.5)		
3	1629(24.0)	543(29.7)			497(29.5)	499(29.7)		
4	3705(54.5)	942(51.5)			909(54.0)	892(53.0)		
5	992(14.6)	121(6.6)			117(7.0)	119(7.1)		
**Hypertension**	1703(25.1)	552(30.2)	<0.001	11.60	481(28.6)	495(29.4)	0.509	1.83
**Diabetes mellitus**	840(12.4)	314(17.2)	<0.001	13.67	248(14.7)	273(16.2)	0.180	4.11
**Tuberculosis**	103(1.5)	65(3.6)	<0.001	13.03	51(3.0)	49(2.9)	0.834	0.70
**Hepatitis carrier**	179(2.6)	51(2.8)	0.704	0.99	42(2.5)	50(3.0)	0.365	2.92
**Cancer**	1267(18.6)	600(32.8)	<0.001	32.89	526(31.3)	516(30.7)	0.611	1.29
**Pulmonary disease**	256(3.8)	178(9.7)	<0.001	23.97	146(8.7)	139(8.3)	0.628	1.49
**Cardiovascular disease**	533(7.8)	198(10.8)	<0.001	10.30	175(10.4)	172(10.2)	0.852	0.59
**Renal disease**	279(4.1)	120(6.6)	<0.001	10.97	100(5.9)	110(6.5)	0.440	2.46
**Cerebrovascular disease**	289(4.3)	127(7.0)	<0.001	11.75	106(6.3)	107(6.4)	0.940	0.24
**Organ transplantation**	67(1.0)	39(2.1)	<0.001	9.28	34(2.0)	34(2.0)	>0.999	0
**Central catheterization**	5(0.1)	21(1.2)	<0.001	13.83	3(0.2)	5(0.3)	0.317	2.44
**Mechanical ventilation**	78(1.2)	57(3.1)	<0.001	13.67	36(2.1)	34(2.0)	0.796	0.83
**Continuous renal replace**	0(0.0)	0(0.0)		0	0(0.0)	0(0.0)		0
**Inotropic use**	11(0.2)	39(2.1)	<0.001	18.59	11(0.7)	12(0.7)	0.827	0.72

Variables are shown as mean ± standard deviation or number (percentage).

Abbreviations: ASD, absolute standardized difference; KTAS, Korean Triage and Acuity Scale

### Main analysis

After matching, the mean length of stay in the ED (ED LOS) for hospitalized patients was 406.5 min (standard deviation [SD], 237.9 min) for those in the isolation care unit and 360.2 min (SD, 226.4 min) for those in the non-isolation care unit. The mean difference between the groups was 44.0 min (95% CI, 26.4 min to 58.7 min), which indicated non-inferiority of the isolation care unit (p = 0.0037). However, the bootstrap validation did not show non-inferiority between the mean ED LOS for hospitalized patients in the isolation care unit and those in the non-isolation care unit (95% CI, 26.4 min to 61.4 min).

The mean difference between the ED LOS for patients discharged from the ED was 139.9 min (95% CI, 123.8 min to 153.3 min, [124.0 min to 156.2 min in the bootstrap validation]), which did not show non-inferiority of the isolation care unit (p>0.999).

**Fig 3:**
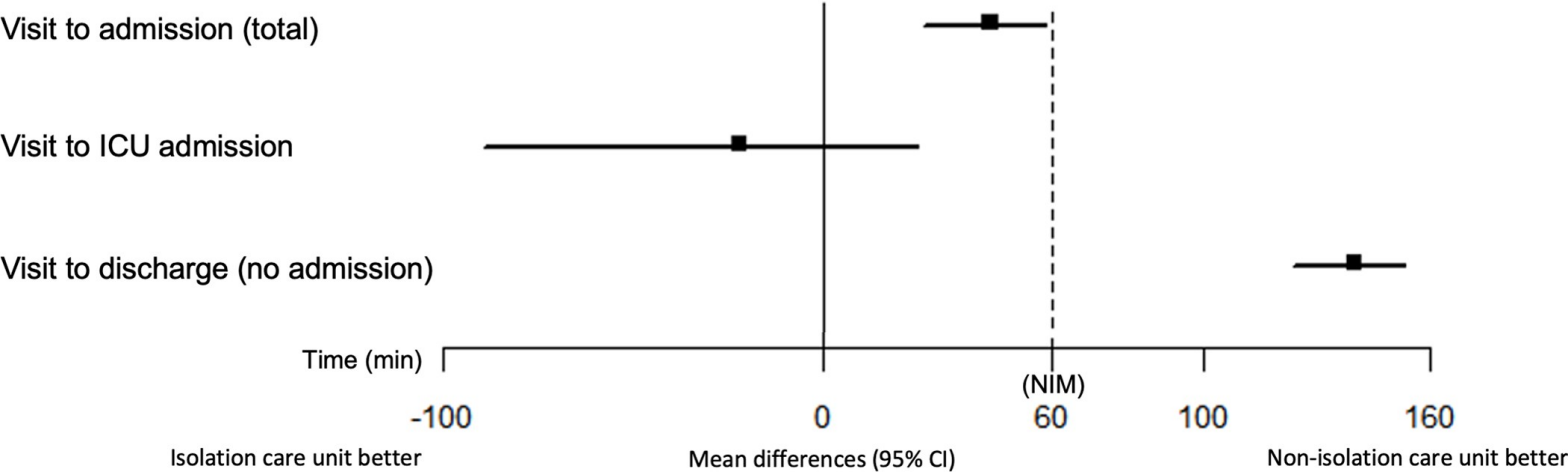
Mean differences between length of stay in the emergency department, ICU: Intensive care unit.

For patients who were admitted into the ICU, the mean ED LOS was 369.4 min (SD, 248.6 min) for patients in the isolation care unit and 276.5 min (SD, 204.3 min) for those in the non-isolation care unit. The mean difference in ED LOS between the isolation and non-isolation care units was -22.0 min (95% CI, -89.4 min to 24.7 min, [-88.6 min to 43.3 min in the bootstrap validation]), which indicated non-inferiority (p = 0.008) (Fig 3).

### Subgroup analysis

**Fig 4:**
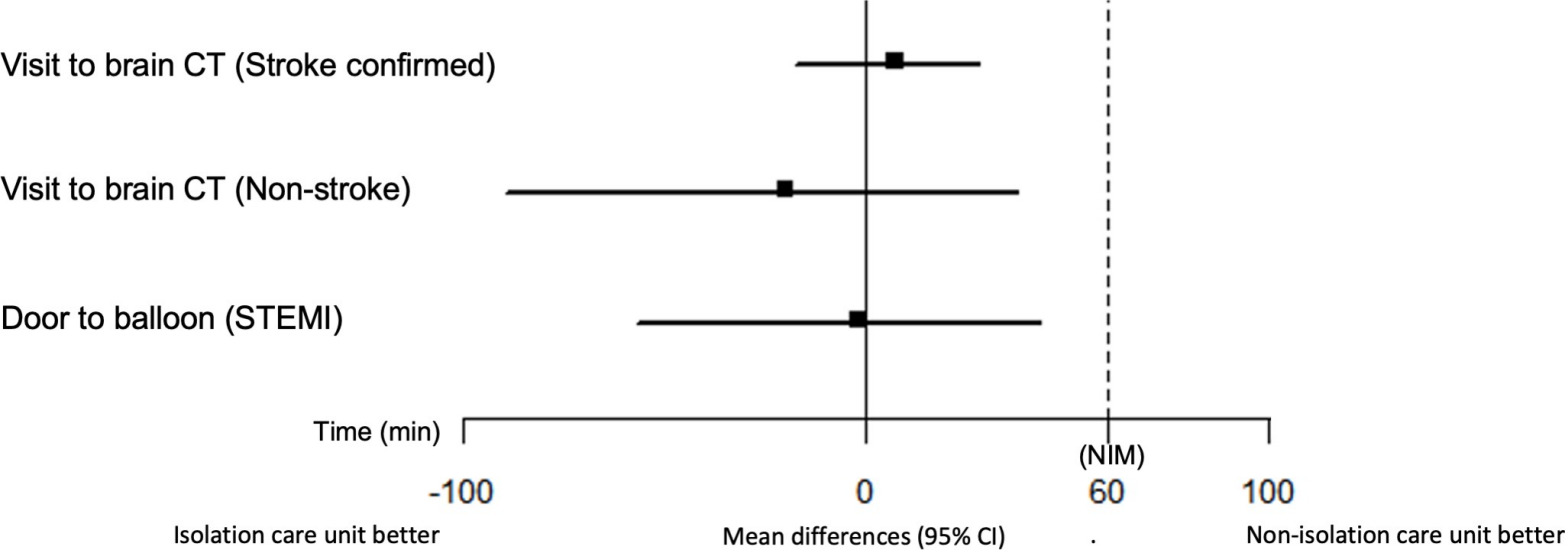
Mean differences between the door-to-brain computed tomography times of patients suspected to have stroke and the door-to-balloon times of patients suspected to have ST-segment elevation myocardial infarction, CT: Computed tomography.

For door-to-brain CT scan times of 269 patients with neurologic deficits that were stroke-related symptoms, the mean difference between the isolation and non-isolation care units was 7.4 min for patients with confirmed stroke (95% CI, -17.3 min to 28.1 min) (p = 0.013) and -20.1 min for discharged patients (95% CI, -89.5 min to 38.0 min) (p = 0.012), which indicated non-inferiority of the isolation care unit. Moreover, for 42 patients who underwent coronary angiography, the mean difference between the door-to-balloon times of the isolation and non-isolation care units was -2.1 min, which indicated non-inferiority of the isolation care unit (95% CI, -56.8 min to 43.4 min) (p<0.001) (Fig 4). In the bootstrap validation, the mean differences between the isolation and non-isolation care units for door-to-brain CT time in patients with confirmed stroke, door-to-brain CT time in discharged patients with symptoms related to stroke, and the door-to-balloon time in patients that underwent coronary angiography indicated non inferiority of the isolation care unit (95% CI, -29.07 min to 31.09 min, -81.66 min to 51.34 min, and -39.18 min to 41.06 min, respectively).

## Discussion

In this study, we described a novel and efficient method of care to prevent the degradation of the quality of diagnosis and treatment of critical diseases during the COVID-19 pandemic. This study showed that essential medical practice, including emergency treatment for non-COVID-19 patients who visited the ED, has not been compromised by our system. From January to February 2020, immediately after the COVID-19 outbreak, the ED and infection control team of the hospital worked together to adjust the infrastructure of the ED to fit the post-pandemic era. Therefore, an efficient method was established to maintain requisite ED functions while simultaneously treating suspected COVID-19 and other conditions.

After the COVID-19 outbreak, the number of patients who visited our ED decreased significantly; this occurrence was consistent with the global trends. The issues caused by the lack of proper distribution of medical resources to patients who visit the hospital have been identified in previous studies [14, 15]. Bjornsen et al. showed that the number of patients who visited the ED reduced to 61% in weeks 2–10 of 2020; however, the number of patients with potentially infectious diseases increased, and those patients stayed 2 h longer than other patients [16]. Wang et al. reported a decrease in the number of patients who were transferred to the ED from the outpatient fever clinic and an increase in the amount of time patients spent in the fever clinic (55 min vs 203 min, p<0.001), compared with that in the pre-pandemic era. In this study, the total LOS before and after the COVID-19 pandemic were 22 and 442 min, respectively (p<0.001). The number of in-hospital deaths increased from 9 of 29 to 21 of 38 for critically ill patients [17]. Barrett et al. simulated scenarios of hospital health care capacity and showed that in a worst-case scenario, >13,000 patients would die while waiting for needed resources [18]. For these reasons, methods such as infection control, risk management to prevent collateral damages, surge planning, and staff protection and wellness were emphasized in previous studies as techniques to help combat the challenges of the COVID-19 pandemic in hospitals [19, 20].

During the 3-month study period, a total of 6,420 patients were examined for COVID-19; six of them tested positive for COVID-19. Notably, there was no cross-infection case within the ED of our hospital. Disposition and admission of patients were established at the same level regardless of whether the patient was admitted and treated in an isolation care unit due to suspected infection or not. However, the ED LOS for patients who were not admitted into the isolation care units did not show non-inferiority compared with that of patients in the general zone. This result implies that the medical resources were properly distributed to patients who needed hospitalization for additional care, rather than to the patients who did not need hospitalization. However, this result might have been overestimated since patients who stayed in the ED until confirmation of their negative COVID-19 test result before transfer to a nursing care hospital or long-term care hospital for conservative treatment after discharge were included in the dataset.

In terms of contributing to the global burden of disease with conditions such as ischemic stroke and ischemic heart disease, several studies have shown that the COVID-19 pandemic has influenced the management of critical disease and has been associated with a delay in proper treatment, thereby leading to poor outcomes [21–25]. In patients with underlying medical conditions, in particular, COVID-19 may cause severe health deterioration [26–29]. Compared with the findings in our study, Katsanos et al. noted an increase in the median door-to-CT times for patients who received intravenous tissue plasminogen activator (19 min, interquartile range [IQR]: 14 min to 27 min vs. 13 min, IQR: 9 min to 17 min, p = 0.008) and endovascular therapy (20 min, IQR: 15 min to 33 min vs. 11 min, IQR: 5 min to 20 min) (p = 0.035) [30]. Moreover, according to Siegler, the COVID-19 pandemic has been shown to result in a significant delay in administration of intravenous thrombolysis for patients with acute ischemic stroke, since patients with stroke during the COVID-19 pandemic were at lower odds of undergoing thrombolysis within 60 min of arrival (odds ratio [OR] 0.61 [95% CI, 0.38 to 0.98]; p = 0.04) [31]. In a French national study conducted in 2019, a delay in mechanical thrombectomy for acute ischemic stroke (mean, 144.9±86.8 min vs 126.2±70.9 min; p<0.001) was associated with mortality [32]. Furthermore, for patients with ischemic heart disease, COVID-19 has been associated with a significant increase in door-to-balloon and total ischemia times, which may result in increased mortality [33, 34]. In another study, the duration from arrival to first medical contact for patients who directly visited the ED was increased (238 min vs 450 min; p = 0.04) [35]. However, in this study, the door-to-balloon and door-to-brain CT times of patients who had risk factors for COVID-19 showed non-inferiority compared with those of patients who did not meet the screening criteria. The consistency of our study results in 10,000 random bootstrap sample runs indicated the robustness of our findings. Therefore, this implies that the medical personnel handled the resources and clinical emergency situations safely despite the possibility of infection.

From an economic perspective, only minor expenditures were incurred as initial costs, such as acrylic board installation and patient waiting room renovation, since we were already equipped with increased distances between beds and laminar-flow air-conditioning before the COVID-19 pandemic. Prolonged hospitalization can lead to generation of additional costs due to healthcare-associated infections, which was shown to be €5,823−€11,840 ($7,453−$15,155) per infected patient in a single hospital [36]. Through incorporation of the methods described in this study, it may be possible to reduce the economic burden on hospitals by preventing cross-infections in the ED and unnecessary prolongation of hospital stay. Moreover, our methods could promote economic savings by maintaining the time from arrival to the ED to the initial CT scan for patients with suspected stroke and door-to-balloon time for patients with STEMI as analogous to the time spent on patients without any infectious symptoms, despite performing screening procedures for patients with symptoms suggesting infectious disease. Costs of productivity loss due to premature death of patients with delayed treatment of critical disease such as myocardial infarction and stroke can be reduced by implementing the cost-benefit analysis, considering the national socioeconomic status of South Korea. The cost of $28 million productivity loss resulting from premature death at productive ages (15–64 years) was estimated assuming the loss of productivity due to death or severe illness in patients of this study, according to sex and age group using the statistical data on employment [37, 38]. Therefore, preventing treatment delay in those with and without infection signs is expected to maintain the regularity of medical service for patients with time-sensitive critical disease, thereby reducing the mortality rate of critical patients due to infectious disease outbreaks and productivity losses.

The allocation of the patients with COVID-19 was determined by the Korea Centers for Disease Control and Prevention and public health centers nationally. Our hospital operated isolation wards for critically ill patients with confirmed COVID-19. Simultaneously, a preemptive isolation ward, in which a suspected infectious patient was admitted, and treatment was rapidly initiated even though COVID-19 was not yet confirmed, was operated in close connection with the ED. A total of 357 patients in the isolation care unit were admitted to the preemptive isolation ward. Once admission to the hospital was decided, an internal medicine physician and an emergency physician communicated closely regarding the medical condition of the patient, and the patient was rapidly moved to the preemptive isolation ward after the evacuation of a path. This strategy plays an important role in the efficient control of in-hospital cross-infections, preventing the shutdown of the hospital. The four-tier system of patient flow and spatial strategy described in this study provided flexibility in the allocation of medical resources to patients who met the screening criteria and those who did not by compartmentalizing the treatment area and assignment of medical staff for dedicated care in each section. Therefore, patients admitted to the hospital through isolation care units were 27.5% of all adult inpatients admitted via the ED, and no case of infection of medical staff and cross-infection between patients occurred during the study period.

The strength of the multi-tiered strategy in this study is that it makes the most of the existing human, physical, and spatial resources by software modifications, which has changed the way patients in the ED are managed, and utilization the negative pressure rooms and isolation areas as treatment spots, and not as a fixed space for staying. Therefore, application of the methods demonstrated in this study to other ED may help in achieving an analogous effect in rapid triage and treatment initiation for not just COVID-19-related symptoms but also for other critical medical conditions, without the consumption of additional spatial/human resources. However, there may be some issues associated with the transformation of the ED. The ED transformation was readily possible in this study since the South Korean government regulated the number of patients in the ED and maintenance of the 2-m distance between the beds after the Middle East respiratory syndrome pandemic experience. Moreover, the number of patients visiting the ED decreased significantly during the COVID-19 pandemic, which allowed for complete utilization of the existing resources. This study presents a way to provide optimal treatment to both suspected and non-infectious patients without losing the basic function of the ED in a situation where screening for unknown infectious diseases is required. However, if the total number of inpatients does not decrease, or in case of a disaster in which a decline in the basic functionality of the ED is inevitable, an expansion of the space and recruitment of additional staffing may be essential.

In this study, we analyzed the changes in the ED, which were appropriately adapted to the infectious disease outbreak and affected the treatment of patients both at risk of infectious diseases and those who were not. To the best of our knowledge, no previous studies have demonstrated the effectiveness of a structural change in the ED on the maintenance of essential functions of the ED and management of patients whose treatments were delayed due to uncertainty of COVID-19 results. Therefore, this study has considerable clinical significance in that these strategies can facilitate the accommodation of patients who need immediate and critical care while awaiting the confirmation of their COVID-19 test results, as well as provide evidence of the effect of protecting patients and medical staff from further infections.

This study has a few limitations. First, due to the retrospective nature of the study, unidentified confounders might have existed despite the propensity score matching performed. Second, since this study was conducted at a single tertiary hospital, further studies on several EDs are warranted. Moreover, as this study was conducted for a relatively short period, the long-term effect of its findings could not be concluded.

## Conclusion

Based on this study, the suggested principles for infection control in the ED are as follows: patients should be screened for infection before entering the ED, contact should be minimized during the medical care process, the best care should be provided to critical patients with suspected infection and non-infectious critical patients, and separate resources should be provided for the care of patients with suspected infection and other critical patients. The COVID-19 pandemic is prolonged and poses certain risks that hinder the maintenance of essential functions in the ED. Flexible handling of a carefully and creatively planned ED plays a key role in dispelling concerns regarding the quality of medical care as well as reducing the likelihood of the spread of infection.
